# The Digital Twin Modeling of Facial Pores Associating Skin Laxity

**DOI:** 10.1111/jocd.71232

**Published:** 2026-10-20

**Authors:** Jin Ho Kim

**Affiliations:** ^1^ V&Co Cooperation Seoul Korea; ^2^ V‐Plastic Surgery Daegu Korea

**Keywords:** digital twin, facial pore, High intensity focused ultrasound, in‐silico simulation, Microneedle radiofrequency, skin rejuvenation

To the Editor,

Enlarged facial pores associated with skin laxity present a prevalent aesthetic challenge. Clinically, senescent pores do not expand uniformly but change with aging from circular openings into elongated, elliptical furrows aligned with Langer's lines [1]. While targeted interventions utilizing microneedle radiofrequency (RF), high‐intensity focused ultrasound (HIFU) [2], and superficial hyaluronic acid (HA) biostimulators [3] demonstrate significant efficacy, a unified biomechanical rationale explaining this asymmetrical yielding and how disparate modalities resolve this distinct pathology remains unveiled.

To bridge this gap, we developed an in silico poro‐visco‐diffusive (PVD) continuum digital twin. By coupling localized thermal strain tensors and Donnan osmotic swelling pressures, the simulation mathematically renders 2D surface morphodynamics and 3D high‐frequency ultrasound (HFUS) echogenicity (Detailed mathematical formulations are provided in the Supporting Information S1–S4).

In youthful skin, structural equilibrium relies on an internal hydraulic restriction: bound‐water turgor neutralizes the Kirsch hoop stress exerted by Langer's lines. Our simulation demonstrates that chronological matrix degradation and loss of turgor precipitate an entropic transition. Bereft of internal compressive pressure, the infundibulum could not endure macroscopic skin tension associated with skin laxity, mathematically emerging as anisotropic elliptical furrows (Figure 1). Concurrently, structural fragmentation and unbound fluid accumulation physicalize a subepidermal low echogenic pixel (SLEP) halo around the dilated ostium, reflecting a localized drop in shear modulus and acoustic impedance within the peri‐infundibular zone. This computational reconstruction partially mirrors the cross‐sectional geometry of the aged follicular opening; however, without considering complex pilosebaceous adnexa such as sebaceous gland, apocrine gland, and arrector pili muscle.

**FIGURE 1:**
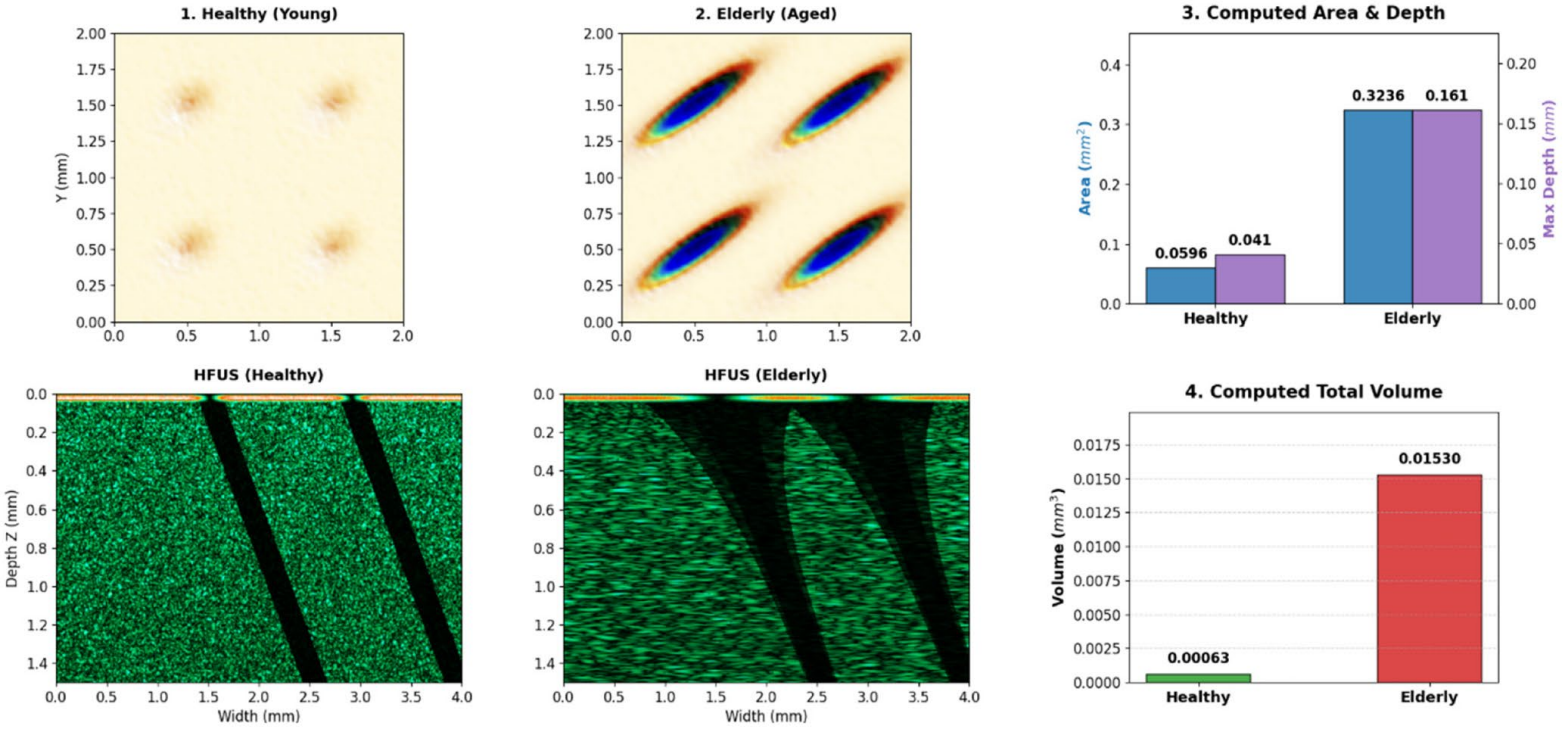
In silico modeling of young and aged facial pore including morphodynamics, high‐frequency ultrasound echogenicity, and morphometric data.

Crucially, extracting tri‐axial morphometric data (Pore area, Depth, and volume) from our model uncovers a profound “Area‐Depth Dissociation” that differentiates the reparative kinetics of therapeutic modalities (Figure 2). When simulating intradermal biostimulators, deep reticular injections (> 1.0 mm) dissipate osmotic forces into structurally stable, low‐compliance zones, failing to modify surface topography. Conversely, superficial HA (0.3 mm) leverages thermodynamic confinement within the highly compliant papillary dermis. This restricts hydro‐distension precisely to the yielded infundibular neck, generating localized radial compression that actively elevates the atrophic pore floor, optimally minimizing Max Pore Depth and Total Volume [3]. This demonstrates that fluid phase replenishment must be micro‐anatomically targeted to overcome gravity‐induced invagination.

**FIGURE 2:**
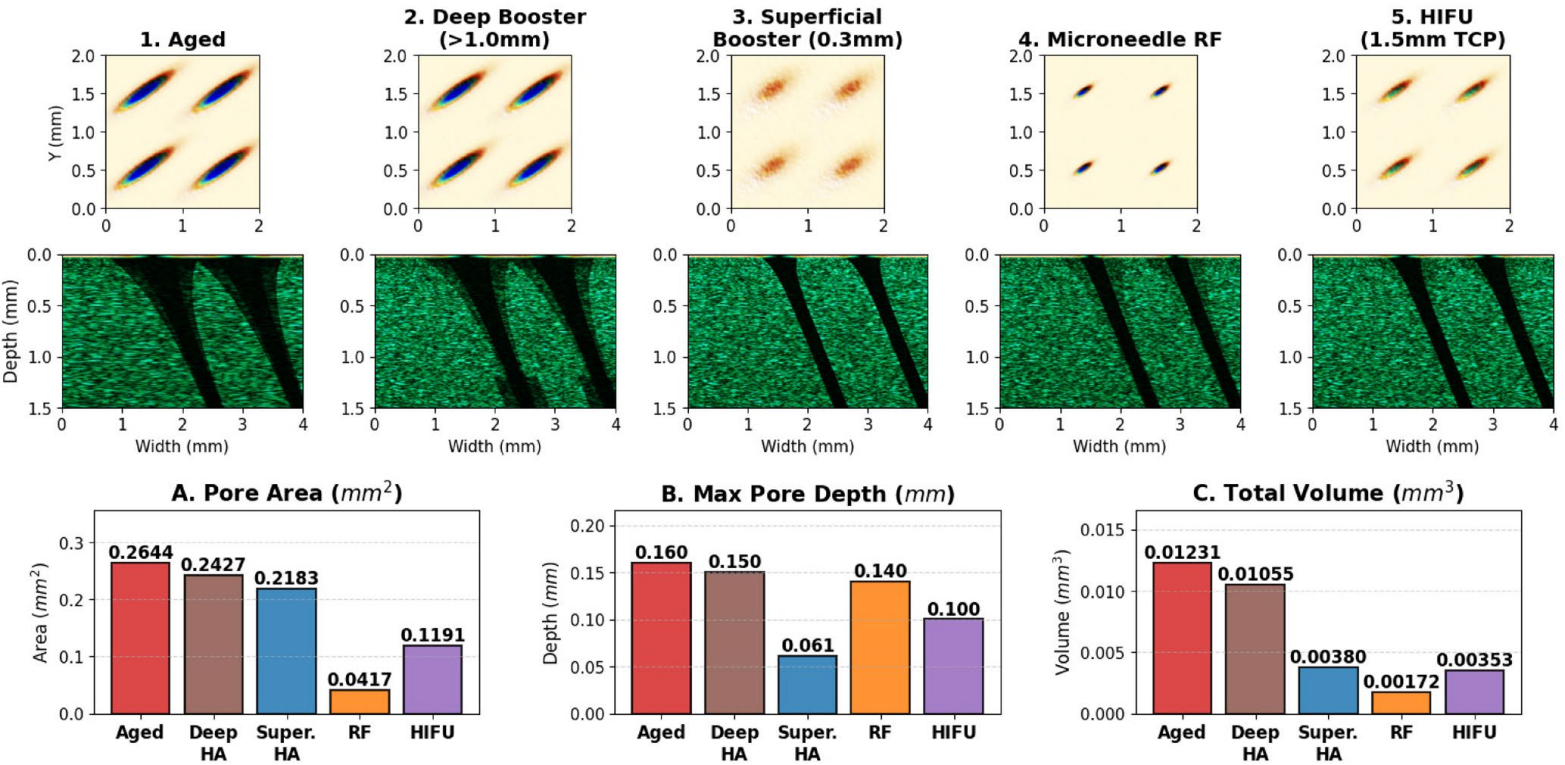
In silico modeling of facial pore changing after interventions including skin booster, microneedle radiofrequency (RF), and high‐intensity focused ultrasound (HIFU). (A) Pore Area, the projected area below the threshold plane (B) Max Pore Depth, the maximum vertical distance from the threshold plane to the pore floor (C) Total Volume, the integrated void volume beneath the threshold plane within the 2.0 × 2.0 mm region of interest.

In the energy‐based domain, the model highlights distinct spatial boundary conditions for thermal strain. Microneedle RF induces localized cross‐linking of the immediate peri‐follicular collagen lattice. This localized lateral cinching successfully restores structural hoop stress, narrowing the topological Area into a slit, yet fails to elevate the atrophic floor due to the lack of volume‐expanding fluid phase transitions. In contrast, HIFU bypasses the superficial orifice entirely, depositing focal thermal coagulation points (TCP) at 1.5 mm. Viscoelastically, these deep macro‐anchors establish a geometric “Tension Shield.” By absorbing far‐field skin sagging vectors and offloading tensor loads from the superficial epidermis, this shield allows the dilated infundibulum to undergo passive elastic recoil, yielding balanced, indirect improvements in both Area and Depth [2]. Thus, energy‐based devices restructure the mechanical boundary conditions without altering hydration states.

We acknowledge that pore enlargement is multifactorial. This HFUS‐based model specifically isolates passive senescent yielding associated with skin laxity; active seborrheic dilatation was excluded, as HFUS is inherently limited in mathematically differentiating microscopic lipid‐rich sebum plugs from purely aqueous voids. Integrating active neuro‐endocrine sebum dynamics remains a subject for future optical simulations.

In summary, our digital twin unequivocally proves that monotherapies act unilaterally on specific topological dimensions; therefore, the true spatial obliteration of the senescent pore is not merely a superficial repair, but an absolute biophysical rollback to youthful tensegrity that fundamentally mandates the synergistic superimposition of targeted thermo‐mechanical shrinkage and chemo‐thermodynamic inflation.

## Author Contributions

Dr. Jin Ho Kim contributed to data curation, analysis, investigation, and writing the original draft.

## Ethics Statement

The author has nothing to report.

## Conflicts of Interest

The author declares no conflicts of interest.

## Supporting information


**Supporting Information S1:** Poro‐visco‐diffusive (PVD) continuum framework.
**Supporting Information S2:** Pathological transition skin laxity.
**Supporting Information S3:** Mathematical modeling of therapeutic modalities.
**Supporting Information S4:** Calculation of pore area, depth, and volume.

## Data Availability

The data that support the findings of this study are available from the corresponding author, AC upon reasonable request.

## References

[1] S. I. Jang , E. J. Kim , and H. K. Lee , “A Method of Evaluating Facial Pores Using Optical 2D Images and Analysis of Age‐Dependent Changes in Facial Pores in Koreans,” Skin Research and Technology 24, no. 2 (2018): 304–308, 10.1111/srt.12430. 29383766

[2] J. Y. Park , S. Youn , W. Hong , et al., “Treatment Protocol on Using Microfocused Ultrasound With Visualization for Skin Quality Improvement: The Korean Experience,” Plastic and Reconstructive Surgery‐Global Open 11, no. 5 (2023): e5029, 10.1097/GOX.0000000000005029. PMC10219723 37250837

[3] T. Rutnumnoi , P. Palakornkitti , T. Anuntrangsee , et al., “Superficial Intradermal Injections of Cohesive Polydensified Matrix Hyaluronic Acid Fillers for the Improvement of Facial Pores and Skin Quality: A Split‐Face Randomized Study,” Journal of Cosmetic Dermatology 24, no. 5 (2025): e70209. 10.1111/jocd.70209 PMC12042064 40304039

