# Supplementary material for: The Digital Twin Modeling of Facial Pores Associating Skin Laxity
Source: J Cosmet Dermatol. 2026 Oct 20;25(10):e71232. doi: 10.1111/jocd.71232 (PMC13684052; doi:10.1111/jocd.71232)
Supplement: Supplementary file 1 [file JOCD-25-e71232-s001.docx]

**Supporting Information S1. Poro-Visco-Diffusive (PVD) Continuum Framework**

The digital twin models the human skin as a multi-phase, fluid-saturated porous medium governed by poro-viscoelasticity. The total Cauchy stress tensor $\boldsymbol{\sigma}$ within the dermal matrix is formulated by separating the solid matrix stress and the fluid phase pore pressure, satisfying the macroscopic equilibrium equation:

$$\nabla\cdot\boldsymbol{\sigma}=0$$

$$\boldsymbol{\sigma}=\boldsymbol{\sigma}_{matrix}-\alpha p_{f}\boldsymbol{I}$$

where $\boldsymbol{\sigma}_{matrix}$ represents the viscoelastic stress tensor of the collagen-elastin fiber network, $\alpha$ is the Biot-Willis coefficient ($0<\alpha\leq1$), $p_{f}$ is the internal fluid pore pressure (hydraulic turgor), and $\boldsymbol{I}$ is the second-order identity tensor.In youthful skin, the matrix maintains a high shear modulus $G_{0}$ and baseline hydraulic turgor $p_{f,young}$. The geometric boundary condition at the skin surface incorporates the far-field Kirsch hoop stress $\sigma_{\infty}$ generated along Langer’s lines:

$$\sigma_{\infty}=\frac{T_{\infty}}{h}$$

where $T_{\infty}$ is the macroscopic skin tension associated with structural orientation and $h$ is the dermal thickness.

**Supporting Information S2. Pathological Transition Skin Laxity**

In elderly skin (Figure 1), chronological matrix degradation induces an entropic loss of structural integrity, characteristically simulated by an exponential decay of the shear modulus $G(t)$ and a drastic drop in bound-water hydraulic turgor ($p_{f}$ to 0). Bereft of internal hydraulic counter-pressure, the pilosebaceous infundibulum kinetically surrenders to the directional tensor $\sigma_{\infty}$.

The resulting asymmetrical topographic deformation $Z_{surf}\left( x,y \right)$ is mathematically rendered via an anisotropic Gaussian distribution aligned with the rotated axis of Langer's tension ($x_{rot}, y_{rot}$):

$$Z_{surf}\left( x,y \right)=Z_{0}-d_{max}\cdot\exp\left( -\left[ \frac{x_{rot}^{2}}{2\sigma_{x}^{2}}+\frac{y_{rot}^{2}}{2\sigma_{y}^{2}} \right] \right)$$

where $Z_{0}$is the baseline epidermal plane, $d_{max}$ is the maximum pore depth, and $\sigma_{x}$, $\sigma_{y}$ represent the spatial variances along the longitudinal and transverse axes, respectively. Under severe skin laxity, the model sets $\sigma_{x}\gg\sigma_{y}$, computing the distinct elliptical morphology.

To simulate high-frequency ultrasound (HFUS) echogenicity, the localized acoustic impedance $Z_{ac}$ is coupled to the spatial density of the collagen matrix $\rho_{m}$ and the concentration of free, unbound interstitial fluid $C_{free}$:

$$Z_{ac}\left( x,z \right)\propto\left[ \rho_{m}\left( x,z \right)\cdot C_{bound} \right]-\mu_{void}\cdot C_{free}(x,z)$$

where $\mu_{void}$is an empirical attenuation coefficient. Around the collapsed infundibular neck, the breakdown of bound-water complexes leads to localized free-water pooling (increase $C_{free}$), causing a localized collapse in acoustic impedance ($Z_{ac}\left( x,z \right)$ to 0). This mathematically renders the subepidermal low echogenic pixel (SLEP) halo observed in cross-sectional simulations (Figure 1).

**Supporting Information S3. Mathematical Modeling of Therapeutic Modalities**

The clinical interventions analyzed in Figure 2 are simulated by altering either the fluid-phase thermodynamic properties or the solid-phase thermal strain tensors.

1) Chemo-Thermodynamic Plumping (Superficial vs. Deep HA)

Hyaluronic acid injections are modeled by introducing a localized negative charge density, which triggers a massive influx of counter-ions and generates a Donnan osmotic swelling pressure $\Pi_{Donnan}$:

$$\Delta\Pi_{Donnan}=RT\left( \sqrt{c_{f}^{2}+4c_{ext}^{2}}-2c_{ext} \right)$$

where $R$ is the ideal gas constant, $T$ is the absolute temperature, $c_{f}$ is the fixed charge density of the injected HA gel, and $c_{ext}$ is the external mobile ion concentration.

For injecting skin booster in deeper layer (>1.0 mm), the skin booster is deposited in a low-compliance zone where the surrounding matrix possesses high structural stiffness. The boundary conditions confine the volumetric expansion downward, resulting in zero net change at the epidermal surface ($\Delta Z_{surf}\approx0$).

For injecting skin booster superficially (0.3 mm), the skin booster is deployed within the highly compliant papillary dermis. The thermodynamic expansion is localized directly beneath the yielded infundibular neck. The upward radial vector of $\Delta\Pi_{Donnan}$ actively elevates the depressed floor, reducing $d_{max}$without modifying the thermal strain of the matrix ($\boldsymbol{\varepsilon}_{thermal}=0$).

2) Thermo-Mechanical Shrinkage (Microneedle RF and HIFU)

Energy-based devices are simulated by adding a localized thermal strain tensor $\boldsymbol{\varepsilon}_{thermal}$ to the matrix, driven by a temperature field $\Delta T(x,y,z)$ that alters the collagen rest-length:

$$\boldsymbol{\varepsilon}_{thermal}=\int_{t_{0}}^{t_{1}} \alpha_{th}(T)\frac{\partial T}{\partial t}\boldsymbol{I}dt$$

where $\alpha_{th}$ is the structural shrinkage coefficient of collagen, which becomes highly active when T > 60°C.

For microneedle RF, the thermal fields are localized horizontally around the needle tracks in the superficial dermis. This configuration restricts thermal contraction primarily to the horizontal plane ($\varepsilon_{xx}$, $\varepsilon_{yy}$ < 0), executing a lateral cinching that forces the topological Area to decrease drastically while leaving the vertical depth ($d_{max}$) relatively unchanged due to the absence of fluid-phase expansion.

In contrary, High-intensity focused ultrasound deposits discrete thermal coagulation points (TCP) at a deep boundary (z = 1.5 mm). Viscoelastically, these micro-lesions function as fixed mechanical anchors that absorb the far-field tensile load $T_{\infty}$. The boundary condition at the epidermal layer shifts from a tension-loaded state to a mechanically shielded state:

$$\sigma_{\infty,eff}=\sigma_{\infty}-\sigma_{HIFU\_anchor}$$

This deep mechanical offloading allows the superficial tissue surrounding the infundibulum to undergo passive elastic recoil, leading to a balanced reduction in both surface Area and Max Depth.

**Supporting Information S4. Calculation of pore area, depth, and volume**

To generate the comparative bar charts depicted in Figure 1 and Figure 2, the continuous surface profile $Z_{surf}\left( x,y \right)$ is integrated within a defined region of interest $\Omega$ (2.0 mm X 2.0 mm). A fixed topological threshold $Z_{thresh}$ (set at -0.015 mm to simulate standard optical profilometry cut-offs) isolates the pore boundaries via the Heaviside step function $\mathcal{H}$:

$$Pore Area \left( \mathrm{mm}^{2} \right)=\iint_{\Omega} \mathcal{H}\left( Z_{thresh}-Z_{surf}\left( x,y \right) \right)dxdy$$

$$Pore Depth \left( \mathrm{mm} \right)=\max_{\left( x,y \right)\in\Omega} |Z_{thresh}-Z_{surf}\left( x,y \right)|$$

$$Total Volume \left( \mathrm{mm}^{3} \right)=\iint_{\Omega} (Z_{thresh}-Z_{surf}\left( x,y \right)\mathcal{\cdot H}\left( Z_{thresh}-Z_{surf}\left( x,y \right) \right)dxdy$$

These spatial integrations quantitatively validate the "Area-Depth Dissociation," confirming that energy-based lateral contraction and thermodynamic vertical inflation operate on distinct biophysical dimensions.
